# Physical Activities and Parkinson's Disease Progression: A Two‐Sample Mendelian Randomization Study

**DOI:** 10.1111/cns.70296

**Published:** 2025-02-24

**Authors:** Xiaoyue Luo, Cheng Xue, Yongli Pan, Wei Wei, Zhongnan Hao, Zheng Liu, Zijian Zheng, Guohui Lu, Zhipeng Xiao, Meihua Li, Wenqiang Xin

**Affiliations:** ^1^ Department of Neurology, The First Affiliated Hospital, Jiangxi Medical College Nanchang University Nanchang Jiangxi China; ^2^ Jiangxi Key Laboratory of Neurological Diseases, Department of Neurosurgery, The First Affiliated Hospital, Jiangxi Medical College Nanchang University Nanchang Jiangxi China; ^3^ Department of Neurology Shandong Provincial Hospital Affiliated to Shandong First Medical University Jinan People's Republic of China; ^4^ Department of Neurology The Affiliated Hospital of Southwest Jiaotong University & the Third People's Hospital of Chengdu Chengdu Sichuan China; ^5^ Department of Neurology University of Göttingen Medical School Göttingen Germany

**Keywords:** age at onset, Mendelian randomization, Parkinson's disease, physical activity, progression

## Abstract

**Aims:**

To explore the causal relationship between physical activity (PA) and the progression of Parkinson's disease (PD), we conducted a two‐sample Mendelian randomization (MR) analysis.

**Methods:**

Genetic variants were obtained from publicly available genome‐wide association study (GWAS) summary statistics for PA (*N* = 377,000), age at onset (*N* = 28,568), and PD progression (*N* = 4093). Causal estimates were calculated using the inverse variance weighted (IVW) method, with MR‐Egger and weighted median analyses performed to assess the robustness of the results.

**Results:**

Genetically predicted accelerometer‐based overall acceleration average (OAA) was associated with a reduced risk of constipation in PD progression (OR: 0.60, 95% CI: 0.42–0.86, *p* = 5.50 × 10^−3^). Moderate‐to‐vigorous physical activity (MVPA) demonstrated a similar but stronger effect on constipation risk (OR: 0.03, 95% CI: 9.38 × 10^−4^–0.90, *p* = 0.043). Additionally, OAA showed a protective effect on motor experiences of daily living (OR: 0.84, 95% CI: 0.71–1.00, *p* = 0.046). No causal effects were identified for vigorous physical activity (VPA) or the fraction of accelerations exceeding 425 milligravities (FAA) on PD progression.

**Conclusion:**

Our findings suggest a negative causal relationship between PA and PD progression, highlighting the potential role of physical activity in guiding therapeutic strategies for PD management.

## Graphical abstract

This study used Mendelian randomization analysis to explore the causal link between physical activity and various symptoms in the progression of Parkinson's disease. This findings suggested that genetically predicted physical activity engagement exhibited the potential to lower constipation risk and UPDRS2 scores, informing potential PA recommendations for Parkinson's disease management.

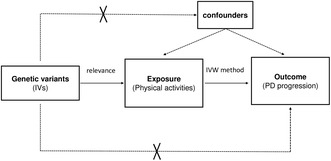

## 1 Introduction

Parkinson's disease (PD) is a prevalent and progressive neurodegenerative disease characterized by a variety of clinical phenotypes encompassing both motor manifestations and non‐motor symptoms such as depression, insomnia, and cognitive dysfunction [1, 2, 3]. Due to the intricate interaction between multiple components like environments, genetic predispositions, and neuropathologies [4, 5], PD has substantial heterogeneity among individuals, including variations in age of onset (AAO) and disease progression [6, 7]. Emerging as a rapidly increasing neurological disease, PD poses a considerable burden on individuals, families, and society [8, 9]. Identifying the modifiable factors that impede PD progression is beneficial for advancing our comprehension of the disease and developing strategies for effective therapeutic interventions, thus improving patients' life and reducing financial expenditure.

In recent years, physical activity (PA) has aroused considerable attention due to its integral role in maintaining brain health [10, 11]. Growing evidence supports the neuroprotective potential of PA, which can be attributed to diverse mechanisms, including the modulation of neurotrophic factor expression, promotion of neuroplasticity, and reduction of inflammation. These multifaceted effects collectively may contribute to delaying brain aging and neurodegenerative pathologies, including PD [12, 13]. However, to date, most clinically based studies investigating the efficacy of PA in PD have been limited by small or single‐center populations, exhibiting inconsistent interventions and varied conclusions [14, 15]. Therefore, large‐scale, multicenter trials are still needed to advance our understanding of the therapeutic potential of PA.

Mendelian randomization (MR), an innovative epidemiological approach, is widely used to investigate causal relationships between exposures and outcomes. Unlike traditional randomized controlled experiments, MR employs genetic variants as instrumental variables (IVs) closely linked to exposures to address causal inquiries [16]. These genetic variants are carried from birth and remain uninfluenced by external environments, thereby mitigating the risk of confounding and reverse causality biases in conventional observational studies [17, 18].

Although previous studies have not established a causal association between PA and the risk of PD using MR analysis [19], the potential relationship between PA and PD progression remains unclear. In this context, we conducted a two‐sample MR analysis to investigate whether PA has a causal effect on PD progression. To comprehensively assess the potential role of PA, we utilized both sets of genetic variants closely linked to PA phenotypes, including traditional self‐reported PA measurements and accelerometer‐measured PA levels obtained from wearable trackers, as reported previously in genome‐wide association studies (GWASs).

## 2 Material and Methods

### 2.1 Ethical Review

All analyses were performed using publicly available data from published literature; therefore, no ethical approval or patient consent was required.

### 2.2 Datasets

We extracted summary statistics for PA from GWAS data based on the UK Biobank (*N* = 377,000) [20]. Significant genetic variants strongly associated with self‐reported and accelerometry‐measured levels of leisure and habitual PA were identified in this research. For self‐reported PA levels, participants completed a touch‐screen questionnaire similar to the International Physical Activity Questionnaire [21]. Based on their responses, two phenotypes were classified: moderate‐to‐vigorous physical activities (MVPA) and vigorous physical activities (VPA). For the assessment of PA through accelerometry, a wrist‐worn accelerometer, Axivity Ax3, was used to monitor daily movement data [22]. Two measures obtained from the wearable device were collected: the overall acceleration average (OAA) and the fraction of accelerations exceeding 425 milligravities (FAA). The cutoff value of 425 mg was chosen according to its correspondence to vigorous physical activity (6 METs), as previously demonstrated [23, 24].

Summary statistics for AAO of PD were obtained from a recently published GWAS, including 28,568 PD patients with European ancestry [25]. Additionally, to investigate the genetically estimated effect of PA on PD progression, we chose the largest publicly available GWAS, which is based on 12 longitudinal PD cohorts with a total of 4093 PD patients [26]. This GWAS involved a meta‐analysis summarizing data from several studies, with varying participant numbers for each symptom.

### 2.3 Genetic Instrument Selection

To identify single‐nucleotide polymorphisms (SNPs) closely linked to PA as IVs, a threshold of *p* < 5E‐08 was chosen for genome‐wide association significance. However, for the phenotype of FAA, a more relaxed criterion (*p* < 5E‐07) was applied due to the limited availability of only two SNPs post‐screening [27]. Subsequently, the PLINK algorithm was employed to verify linkage disequilibrium (LD) (*r* ^2^ < 0.001 and clump distance > 10,000 kb). SNPs displaying LD or significant associations with outcomes were excluded. We also assessed the strength of IVs and excluded SNPs with F statistics below 10 to avoid weak instrument bias. Based on the PhenoScanner V2 database, we further eliminated SNPs associated with potential outcome confounders. Finally, we implemented harmonization to remove ambiguous and palindromic SNPs. Only SNPs meeting all aforementioned requirements were considered valid IVs for MR analysis. Detailed information on the SNPs employed as IVs in the study was shown in Table S1.

### 2.4 Mendelian Randomization Analysis

To evaluate the potential genetic causal effects of PA on the AAO and PD progression, we used the random effects inverse variance weighted (IVW) method as the primary approach for two‐sample MR analysis. This method allows for overdispersion, aggregates the Wald ratio for each SNP with the outcome, and yields an overall estimate of causality. In addition, we employed funnel plots and Cochran's *Q* statistic to identify and evaluate heterogeneity. A significance level below 0.05 in the *Q* statistic indicates substantial heterogeneity, prompting caution in the interpretation of results. To further explore the robustness of our findings and detect possible horizontal pleiotropy, we applied various sensitivity analyses, including leave‐one‐out (LOO) analysis and the MR‐Egger intercept test, as well as MR pleiotropy residual sum and outlier (MR‐PRESSO) analysis. Moreover, to corroborate our MR analysis, we employed weighted median and MR‐Egger regression. The weighted median method gives a credible estimate of the effect even in the presence of up to 50% invalid genetic instruments [28], whereas MR‐Egger regression offers an efficient means to test the null causal hypothesis and can identify unbalanced pleiotropy through the examination of whether the intercept of the exposure‐outcome relationship deviates from zero [29]. However, it is crucial to acknowledge that, due to the inherent properties of MR‐Egger, precision in intercept and causal estimates is generally constrained by limited statistical power [30]. The MR analysis was carried out using R software (version 4.3.2). We utilized the two‐sample MR and forestploter packages to perform statistical analysis and visualize the results. In this exploratory study, findings were not adjusted for multiple testing, and a two‐sided cutoff P‐value of 0.05 was applied for all analyses [31].

## 3 Results

### 3.1 Overall Average Acceleration and PD Progression

**FIGURE 1:**
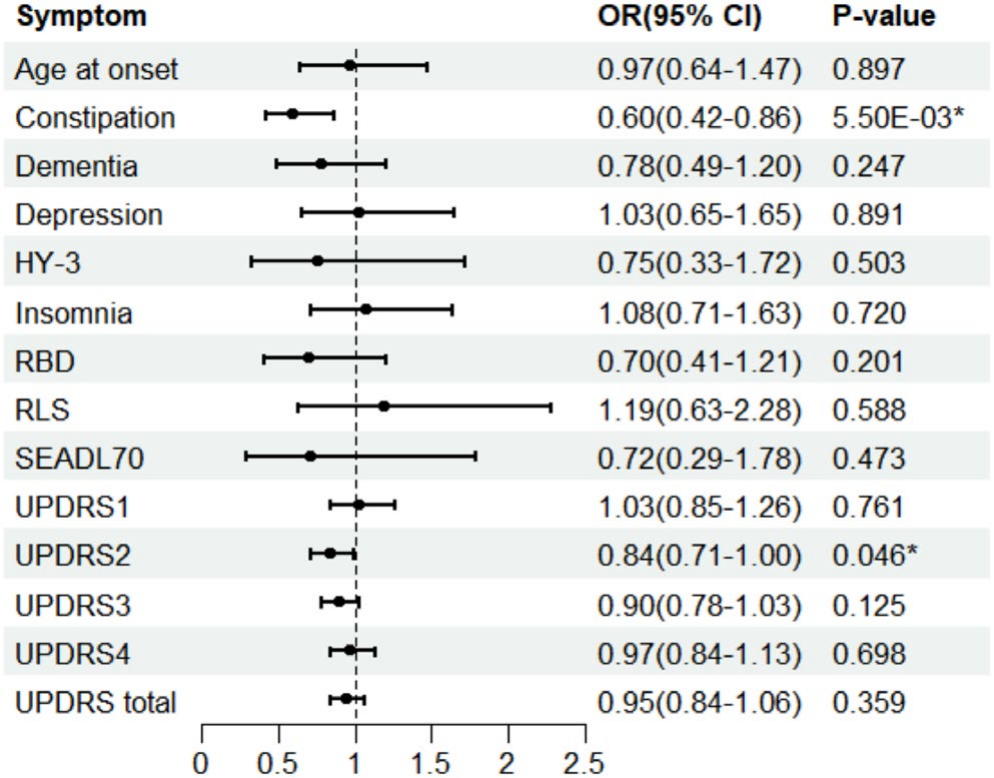
Forest plot of Mendelian randomization analysis to evaluate the genetically predicted causal effects of overall acceleration average on the PD progression. HY‐3, Hoehn‐Yahr scale 3 or greater; RBD, REM sleep behavior disorder; RLS, Restless Legs Syndrome; SEADL70, Schwab and England Activities of Daily Living Scale of 70 or less; UPDRS, Unified Parkinson Disease Rating Scale. An asterisk indicates that the *p* < 0.05.

We investigated the genetically estimated causal association between overall accelerometer‐based physical activity and PD progression via the two‐sample MR approach (Figure 1). IVW analysis indicated a negative relationship between OAA and PD constipation. The odds ratios (ORs) showed a decrement of 0.60 for each standard deviation increase in OAA (95% CI: 0.42–0.86; *p* = 5.50E‐03). This finding was consistently supported by the weighted median method but not by the MR‐Egger analysis (Table 1). Considering the low estimations of MR‐Egger, the examination of horizontal pleiotropy via the MR‐Egger method draws greater attention. In addition, our IVW analysis revealed potential protective effects of OAA on the PD progression, as measured by UPDRS2 scores (OR: 0.84; 95% CI: 0.71–1.00; *p* = 0.046). While results from other MR methods consistently indicated a similar trend, they did not reach statistical significance (Table 1).

**TABLE 1:** Estimated association between OAA and constipation and UPDRS2 in PD progression using different Mendelian randomization methods.

Symptoms of PD progression	MR methods	*p*	OR (95% CI)
Constipation	IVW	5.50E‐03*	0.601 (0.419–0.861)
	MR‐Egger	0.274	0.357 (0.072–1.758)
	Weighted median	5.30E‐03*	0.510 (0.318–0.819)
UPDRS2	IVW	0.046*	0.841 (0.709–0.997)
	MR‐Egger	0.785	0.876 (0.368–2.085)
	Weighted median	0.084	0.838 (0.681–1.031)

Abbreviations: CI, confidence interval; IVW, inverse variance weighted; OAA, overall acceleration average; OR, odds ratio; PD, Parkinson's disease.

* *p* < 0.05 is considered statistically significant.

Subsequently, we conducted pleiotropy and heterogeneity tests to further assess the robustness of the above results. The MR‐Egger intercept consistently demonstrated no significant deviation from zero across all outcomes, indicating the lack of substantial horizontal pleiotropy. Moreover, no detectable heterogeneity was found by Cochran's *Q* test (Table 2). Furthermore, both MR‐PRESSO and LOO analyses failed to identify any outliers. Hence, the observed outcomes were not solely driven by a single genetic instrument (Figures S1 and S2).

**TABLE 2:** Sensitivity test of the Mendelian randomization analysis between OAA and constipation and UPDRS2 in PD progression.

Symptoms of PD progression	Methods	Heterogeneity test		Horizontal pleiotropy		MR‐PRESSO
		Cochran's *Q*	*p*	Egger intercept	*p*	*p* of global test
Constipation	IVW	4.656	0.459	0.144	0.547	0.458
	MR‐Egger	4.202	0.379			
UPDRS2	IVW	0.173	0.996	−0.010	0.929	0.997
	MR‐Egger	0.163	0.983			

Abbreviations: IVW, inverse variance weighted; MR, Mendelian randomization; OAA, overall acceleration average; PD, Parkinson's disease.

### 3.2 Other Physical Activity Phenotypes and PD Progression

Among all other PA phenotypes, including self‐reported PA (MVPA and VPA) and accelerometry‐measured FAA, we only found weak evidence supporting the protective role of MVPA in PD constipation, as indicated by the IVW analysis (OR: 0.03, 95% CI: 9.38E‐04 to 0.90; *p* = 0.043). However, this observed effect failed to reach significance using MR Egger and weighted median methods. Nevertheless, both approaches yielded consistent trends that aligned with the IVW analysis. Additional sensitivity analysis revealed no significant heterogeneity based on the Cochran's *Q* test (*Q* = 8.19, *p* = 0.916). Furthermore, both the MR‐Egger intercept (Egger intercept 0.049, *p* = 0.692) and the MR‐PRESSO global test (*p* = 0.908) failed to demonstrate horizontal pleiotropy (Figure S3).

## 4 Discussion

Using MR analysis, the current study explored the genetically predicted effects of PA on the progression of PD for the first time. Our findings indicated a negative association between two PA phenotypes, namely OAA and MVPA, and the occurrence of constipation in PD progression. Furthermore, OAA appears to exhibit a potential protective effect on PD progression, as reflected by UPDRS2. Our results demonstrate genetic insights into the association between PA and PD progression, thereby enhancing our comprehension of the role of PA in PD.

Among the various symptoms investigated, we discovered the strongest association between OAA and intestinal constipation in PD, which aligns with numerous previous studies [32]. Notably, the protective effect of PA against constipation can persist for several decades [33]. However, the underlying mechanism by which PA mitigates constipation remains uncertain. One plausible explanation is that PA may enhance gastrointestinal motility and accelerate transit time by mechanically stimulating the intestines and triggering the release of pivotal gastrointestinal hormones, thereby helping to prevent and manage constipation [34, 35].

Given the distinctive pathophysiological role of constipation in PD, a prominent non‐motor symptom associated with the autonomic nervous system, postmortem examinations reveal that individuals with constipation are more likely to have Lewy bodies in the locus coeruleus and substantia nigra [36], coupled with a reduction in neuron density in the substantia nigra [37]. On the other hand, PD often manifests with dopaminergic depletion and the presence of Lewy bodies in the myenteric plexus of the colon [38, 39, 40]. Although the precise involvement of constipation in PD development remains uncertain, the brain‐gut axis hypothesis postulates that constipation may potentially facilitate the pathologic process by influencing the intestinal microbiome [41] or through other mechanisms promoting the deposition of α‐synuclein [42]. Therefore, further elucidating the complex interactions between PA and constipation in the neuropathology of PD may be helpful in revealing potential therapeutic targets and improving patient prognosis.

Besides, we noticed a potential protective influence of OAA on the progression of PD as assessed by UPDRS2. In contrast, no such effect was found for other PA phenotypes, including MVPA, VPA, and FAA. UPDRS2 specifically assesses motor experiences in daily living and can serve as a valuable indicator reflecting the quality of life and disability levels of PD patients [43, 44]. Previous studies have shown the improvement of functional movements and quality of life among individuals with PD through a variety of PA phenotypes, including aerobic exercise, dance, Tai Chi, and yoga [45]. However, contrasting findings indicate that certain exercise modalities may not yield the anticipated benefits. Specifically, activities like boxing and cycling have been reported to lack efficacy in improving mobility for PD patients [46]. Furthermore, the influence of anaerobic exercise, as well as combined anaerobic and aerobic training, on the quality of life of PD patients appears to be nonsignificant [47]. Combined with our finding, high‐intensity exercise may not benefit PD patients from progression. Since such an association was only identified through the IVW method in the current study, further exploration of the association between PA and UPDRS2 scores in PD progression is still required. Although we utilized the largest available GWAS summary statistics of PA, we did not classify PA phenotypes in detail, limiting our ability to accurately evaluate the impact of various types of exercise on UPDRS2 scores. Future studies should focus on expanding sample sizes and providing comprehensive classifications of different exercise types, including chronic exercises, to better understand their effects on PD progression.

Regarding other symptoms, we did not find significant associations between PA and the presence of these symptoms in PD progression, despite documentation of specific symptoms in previous research. This may be attributed to the diverse pathogenic mechanisms underlying different PD symptoms. However, we cannot rule out that the sample size was insufficient, thus limiting the ability to detect weaker causal associations. Cohort studies with larger samples are needed for further validation.

However, some limitations should be considered when interpreting the results. Firstly, given its higher statistical power in comparison with other approaches, particularly MR‐Egger, IVW is commonly employed as the primary screening tool for detecting potentially significant outcomes [48]. However, IVW may introduce bias under conditions of horizontal pleiotropy. In contrast, MR‐Egger allows imbalances or directional biases in the horizontal pleiotropic effects of SNPs, providing valuable insights for complementing [48]. Consistent with most MR analyses, we emphasized the importance of maintaining a uniform beta direction across all MR methods [49]. Secondly, MR analysis extrapolates causal hypotheses by using the random assignment of genetic variants. Although we excluded SNPs related to confounders in our analysis, the presence of other unknown confounding factors may still influence the results. Thirdly, the exposure data obtained from wrist‐worn accelerometers, limited to a one‐week duration, may not be sufficient to illustrate routine or lifelong patterns of PA. Additionally, due to the limited information available on the PA phenotypes within the database, further subdivision of the data into specific exercise modalities was not feasible. According to the current published literature, different exercise methods, not just exercise intensity, may be associated with PD progression.

## 5 Conclusion

Based on large‐scale genetic summary data, we used MR analysis to investigate the causal influence of PA on PD progression. The genetically predicted engagement in PA exhibited the potential to reduce the risk of constipation and lower the UPDRS2 scores in individuals with PD. These findings could enhance our comprehension of the role of PA in PD progression and contribute to the development of PA prescribing recommendations for patients with PD.

## Author Contributions

Xiaoyue Luo: data collection, statistical analysis, conceptualization, and manuscript writing; Wei Wei, Yongli Pan, and Zijian Zheng: data collection and analysis of imaging data. Zheng Liu, Zhipeng Xiao, Meihua Li, and Guohui Lu: conceptualization of the paper. Wenqiang Xin: conceptualization and manuscript writing. Cheng Xue and Zhongnan Hao: Data analysis and manuscript writing.

## Conflicts of Interest

The authors declare no conflicts of interest.

## Supporting information


Data S1.

## Data Availability

The data that support the findings of this study are available in GWAS catalog at https://www.ebi.ac.uk/gwas/downloads/summary‐statistics. These data were derived from the following resources available in the public domain: Parkinson disease age at onset GWAS, https://pdgenetics.org/resources GWAS of Parkinson's disease clinical biomarkers, https://pdgenetics.shinyapps.io/pdprogmetagwasbrowser/.
