# Supplementary material for: Physical Activities and Parkinson's Disease Progression: A Two‐Sample Mendelian Randomization Study
Source: CNS Neurosci Ther. 2025 Feb 24;31(2):e70296. doi: 10.1111/cns.70296 (PMC11848732; doi:10.1111/cns.70296)
Supplement: Supplementary file 1 — Data S1. [file CNS-31-e70296-s001.docx]

SUPPLEMENTARY MATERIALS

Physical activities and Parkinson’s disease progression: a two-sample mendelian randomization study

Xiaoyue Luo^1#^, Cheng Xue^2#^, Yongli Pan^3^, Wei Wei^4^, Zhongnan Hao^5^, Zheng Liu^2^, Zijian Zheng^2^, Guohui Lu^2^, Zhipeng Xiao^2^, Meihua Li^2^, Wenqiang Xin^2*^

^1^Department of Neurology, The First Affiliated Hospital, Jiangxi Medical College, Nanchang University, Nanchang, Jiangxi, China.

^2^Jiangxi Key Laboratory of Neurological Diseases, Department of Neurosurgery, The First Affiliated Hospital, Jiangxi Medical College, Nanchang University, Nanchang, Jiangxi, China.

^3^Department of Neurology, Shandong Provincial Hospital Affiliated to Shandong First Medical University, Jinan, People's Republic of China.

^4^Department of Neurology, the Affiliated Hospital of Southwest Jiaotong University & The Third People’s Hospital of Chengdu, Chengdu, Sichuan, China.

^5^Department of Neurology, University of Göttingen Medical School, Göttingen, Germany.

^#^These authors contribute equally to this work and are considered co-first authors.

^*^The author is recognized as corresponding authors for this study.

*Corresponding authors:

Dr. Wenqiang Xin;

Jiangxi Key Laboratory of Neurological Diseases, Department of Neurosurgery, The First Affiliated Hospital, Jiangxi Medical College, Nanchang University, Nanchang, Jiangxi, China.


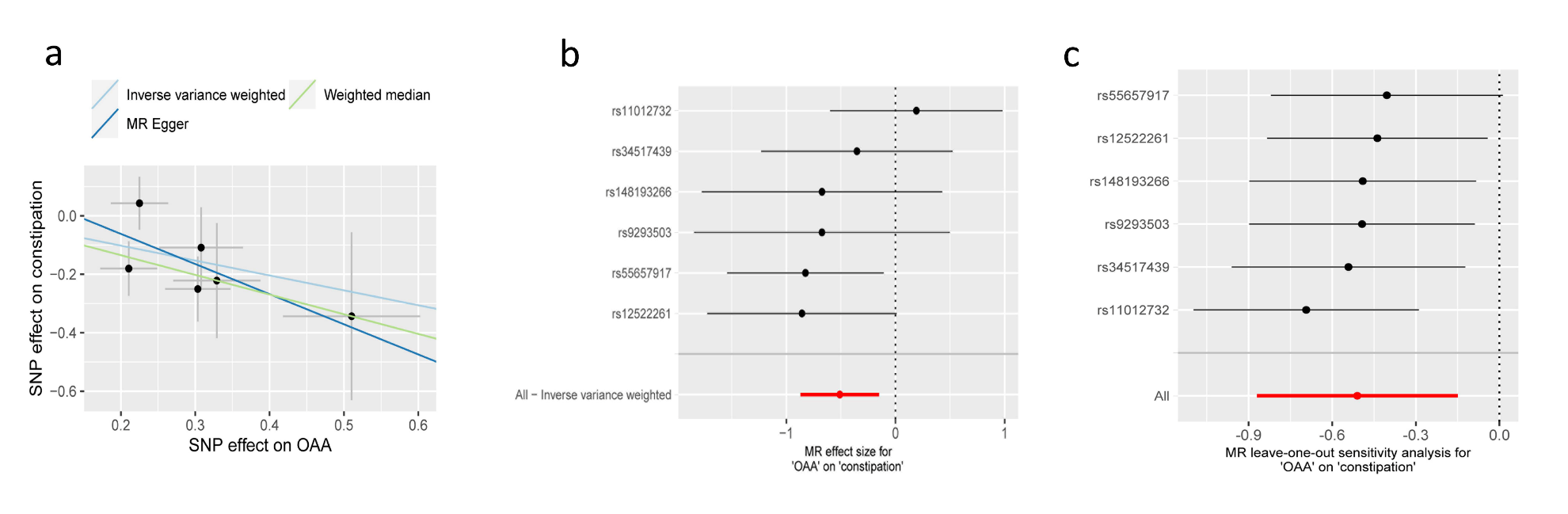


Figure S1. Scatter plot (a), forest plot (b), and leave-one-out analysis (c) illustrating the causal effect of OAA on PD constipation. Abbreviations: OAA, overall acceleration average; PD, Parkinson's disease; SNP, single nucleotide polymorphism.


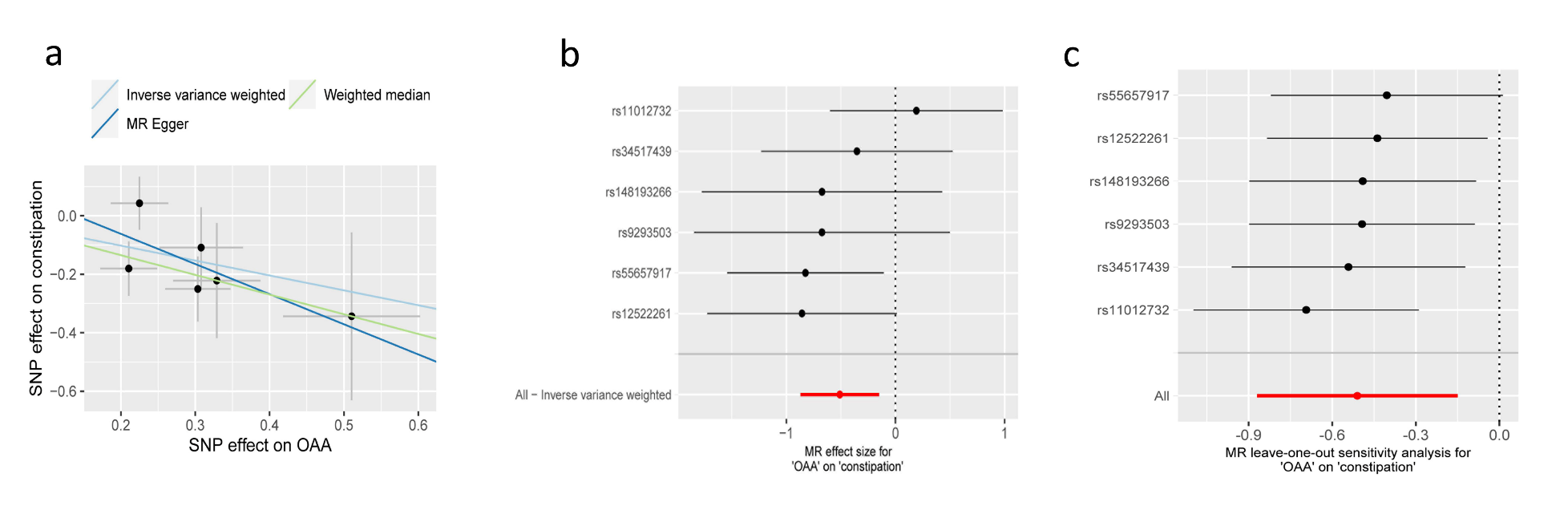


Figure S2. Scatter plot (a), forest plot (b), and leave-one-out analysis (c) illustrating the causal effect of OAA on the UPDRS2 scores of PD progression. Abbreviations: OAA, overall acceleration average; PD, Parkinson's disease; UPDRS2, Unified Parkinson's Disease Rating Scale Part II; SNP, single nucleotide polymorphism.


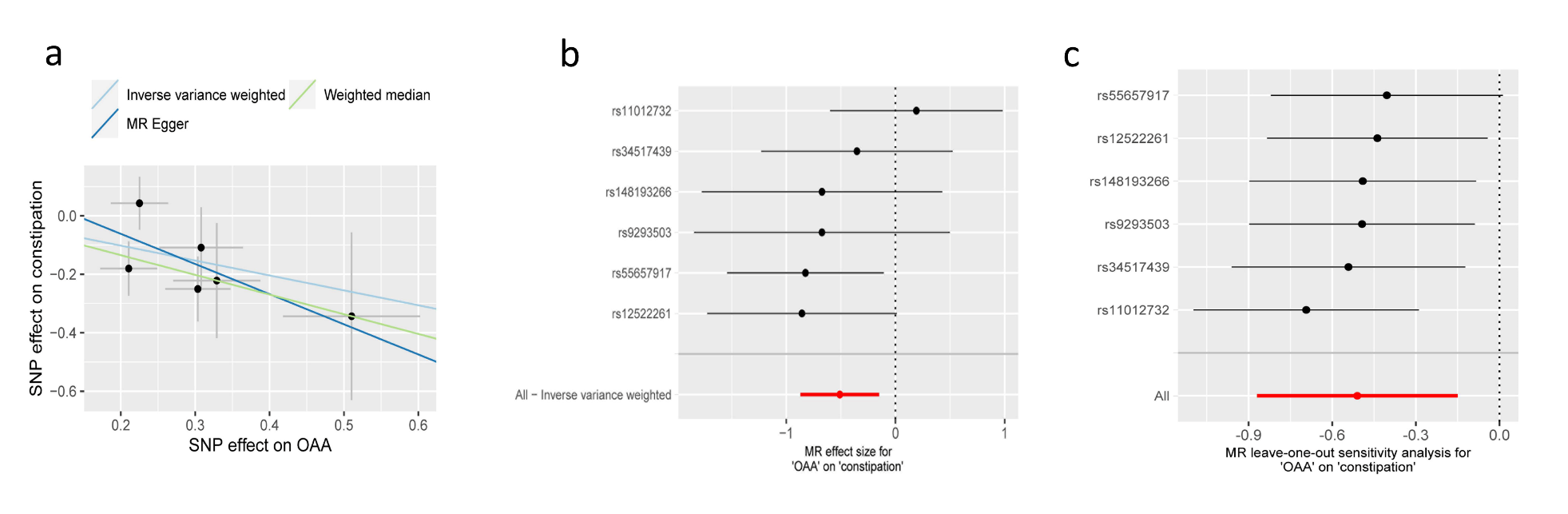


Figure S3. Scatter plot (a), forest plot (b), and leave-one-out analysis (c) illustrating the causal effect of MVPA on PD constipation. Abbreviations: MVPA, moderate-to-vigorous physical activities; PD, Parkinson's disease; SNP, single nucleotide polymorphism.

Table S1: Summary characteristics of selected genetic variants associated with physical activity phenotypes used as instrumental variables.

| PA phenotypes | SNP | EA | OA | EAF | b | se | P | Sample size |
| --- | --- | --- | --- | --- | --- | --- | --- | --- |
| MVPA | rs2942127 | G | A | 0.175 | 0.016 | 0.003 | 3.30E-08 | 377234 |
| MVPA | rs1974771 | G | A | 0.900 | -0.021 | 0.004 | 6.58E-09 | 377234 |
| MVPA | rs2114286 | A | G | 0.466 | -0.012 | 0.002 | 3.34E-08 | 377234 |
| MVPA | rs877483 | T | C | 0.433 | 0.012 | 0.002 | 4.04E-08 | 377234 |
| MVPA | rs2035562 | A | G | 0.328 | -0.014 | 0.002 | 3.87E-09 | 377234 |
| MVPA | rs1972763 | C | T | 0.342 | 0.013 | 0.002 | 3.29E-08 | 377234 |
| MVPA | rs77742115 | T | C | 0.862 | -0.018 | 0.003 | 9.59E-09 | 377234 |
| MVPA | rs2854277 | C | T | 0.917 | 0.032 | 0.005 | 2.59E-10 | 377234 |
| MVPA | rs1186721 | G | A | 0.684 | -0.013 | 0.002 | 4.36E-08 | 377234 |
| MVPA | rs921915 | T | C | 0.412 | -0.014 | 0.002 | 5.66E-10 | 377234 |
| MVPA | rs1043595 | G | A | 0.717 | 0.014 | 0.002 | 4.30E-09 | 377234 |
| MVPA | rs7804463 | T | C | 0.530 | 0.015 | 0.002 | 1.19E-11 | 377234 |
| MVPA | rs2988004 | T | G | 0.558 | -0.013 | 0.002 | 4.09E-09 | 377234 |
| MVPA | rs7326482 | G | T | 0.385 | -0.013 | 0.002 | 1.61E-08 | 377234 |
| MVPA | rs10145335 | G | A | 0.749 | -0.014 | 0.003 | 2.75E-08 | 377234 |
| MVPA | rs4886868 | T | G | 0.414 | -0.012 | 0.002 | 3.51E-08 | 377234 |
| MVPA | rs12912808 | C | T | 0.851 | 0.018 | 0.003 | 1.66E-08 | 377234 |
| MVPA | rs1921981 | G | A | 0.674 | 0.013 | 0.002 | 3.85E-08 | 377234 |
| VPA | rs6667222 | A | C | 0.748 | 0.009 | 0.002 | 8.73E-09 | 261055 |
| VPA | rs1248860 | G | A | 0.484 | -0.010 | 0.001 | 1.06E-13 | 261055 |
| VPA | rs2764261 | A | G | 0.374 | 0.009 | 0.001 | 1.97E-11 | 261055 |
| VPA | rs328902 | C | T | 0.685 | -0.009 | 0.001 | 5.54E-10 | 261055 |
| VPA | rs13243553 | G | A | 0.608 | 0.009 | 0.001 | 9.03E-11 | 261055 |
| VPA | rs3781411 | C | T | 0.876 | 0.013 | 0.002 | 3.01E-10 | 261055 |
| OAA | rs59499656 | A | T | 0.656 | -0.228 | 0.04 | 2.40E-09 | 91084 |
| OAA | rs55657917 | T | G | 0.78 | -0.303 | 0.04 | 5.00E-12 | 91084 |
| OAA | rs148193266 | A | C | 0.957 | -0.51 | 0.09 | 3.10E-08 | 91084 |
| OAA | rs11012732 | A | G | 0.668 | 0.225 | 0.04 | 5.40E-09 | 91084 |
| OAA | rs9293503 | T | C | 0.888 | 0.329 | 0.06 | 2.10E-08 | 91084 |
| OAA | rs12522261 | G | A | 0.657 | 0.211 | 0.04 | 3.90E-08 | 91084 |
| OAA | rs6775319 | A | T | 0.271 | 0.225 | 0.04 | 3.50E-08 | 91084 |
| OAA | rs34517439 | C | A | 0.879 | 0.308 | 0.06 | 4.40E-08 | 91084 |
| FAA | rs1856329 | A | C | 0.801 | 0.027 | 0.005 | 9.00E-08 | 90667 |
| FAA | rs1668835 | T | A | 0.688 | -0.023 | 0.004 | 3.10E-07 | 90667 |
| FAA | rs80028338 | A | C | 0.795 | -0.028 | 0.005 | 1.50E-07 | 90667 |
| FAA | rs743580 | A | G | 0.51 | 0.025 | 0.004 | 1.30E-09 | 90667 |
| FAA | rs4754194 | C | T | 0.773 | -0.025 | 0.005 | 2.40E-07 | 90667 |
| FAA | rs72633364 | G | A | 0.711 | -0.023 | 0.005 | 4.10E-07 | 90667 |
| FAA | rs62443625 | T | C | 0.767 | -0.026 | 0.005 | 1.40E-07 | 90667 |
| FAA | rs6433478 | T | C | 0.457 | -0.024 | 0.004 | 1.20E-08 | 90667 |

^*^: The SNP associated with PD based on the PhenoScanner V2 database which are excluded in the MR analysis. Abbreviations: PA, physical activity; SNP, single nucleotide polymorphism; EA, effect allele; OA, other allele; EAF, effect allele frequency; se, standard error; MVPA, Self-reported moderate-to-vigorous physical activity; VPA, Self-reported vigorous physical activity; OAA, Overall acceleration average; FAA, Fraction of accelerations > 425 milli-gravities.
